# Mastoid pouch of the sigmoid sinus– an extraordinary anatomical variant

**DOI:** 10.1007/s00276-025-03590-3

**Published:** 2025-02-18

**Authors:** Mugurel Constantin Rusu, Corneliu Toader, Răzvan Costin Tudose

**Affiliations:** 1 Division of Anatomy, Faculty of Dentistry, “Carol Davila” University of Medicine and Pharmacy, Bucharest, 020021 Romania; 2 Division of Neurosurgery, Department 6– Clinical Neurosciences, Faculty of Medicine, “Carol Davila” University of Medicine and Pharmacy, Bucharest, RO-020021 Romania; 3 Clinic of Neurosurgery, “Dr. Bagdasar-Arseni” Emergency Clinical Hospital, Bucharest, RO-041915 Romania

**Keywords:** Temporal bone, Mastoid, Lateral sinus, Tympanic cavity, Semicircular canals

## Abstract

**Purpose:**

The dural sigmoid sinus (SS) is a major surgical landmark. It is aimed to report an extraordinary and clinically significant anatomical variation– the mastoid pouch of the SS, which replaces the mastoid air cells.

**Method:**

The archived angioCT file of a 45 y.o. male was retrospectively studied on planar sections and by three-dimensional volume renderings.

**Results:**

An expansive mastoid pouch of the left SS was found, measuring 1.74 cm in transverse diameter and 2.18 cm in sagittal diameter, replacing the typical mastoid air cell system. This SS pouch extended to the mastoid cortex and was covered by a robust (1.41 cm broad) tegmen sinus sigmoidei. The bony wall between the SS pouch and the mastoid portion of the facial canal was 4.65 mm thick.

**Conclusion:**

The marked venous encroachments into the mastoid region emphasise the necessity for comprehensive preoperative imaging and careful selection of surgical approaches to prevent inadvertent vascular compromise.

**Supplementary Information:**

The online version contains supplementary material available at 10.1007/s00276-025-03590-3.

## Introduction

The dural sigmoid sinus (SS) continues the transverse sinus and courses into the posterior cranial fossa towards the jugular foramen. It courses, therefore, in close relation with the mastoid air cell system (MACS), which, in turn, has a variable morphological pattern. The morphology of the SS is most variable at its junction with the transverse sinus and least variable at the jugular bulb [12]. A dominant SS lies more anterior in the temporal bone than a non-dominant SS [12]. Among the known morphological variations of the SS are the diverticula of the SS [5, 9] and the hypoplastic or aplastic SS [4].

The degree of development of the MACS varies from person to person and depends on genetic, environmental, or pathological circumstances [11]. Older age and the presence of cholesteatoma in the mastoid region contribute to the suppression of MACS development in cases of congenital cholesteatoma, as the cholesteatoma itself inhibits the development of the mastoid air cells [11]. The agenesis of the antrum has been reported either in association with congenital syndromes such as trisomy 13 and mandibulofacial dysostosis or as an isolated anomaly [10].

Material and Method.

The archived angioCT file of a 45 y.o. male was retrospectively studied on planar sections and by three-dimensional volume renderings. The CT examination was performed on a 64-slice CT Somatom Definition As (Siemens), with a rotation time of 0.5 s, using a pitch of 1.2 and collimation of 1.2 mm. The technical parameters were detailed previously [8]. Anatomical variants were documented with Horos v3.3.6 software for macOS (Horos Project, Annapolis, MD, USA) on planar or curved planar sections and by three-dimensional volumetric renderings with modulation of the opacity of the tissues. The principles of the Declaration of Helsinki were used to conduct the research. The Ethics Committee (affiliation #3) approved the study (approval no. 2093/1 March 2022). An extraordinary variant of the sigmoid sinus was found and detailed anatomically.

## Results

**Fig. 1:**
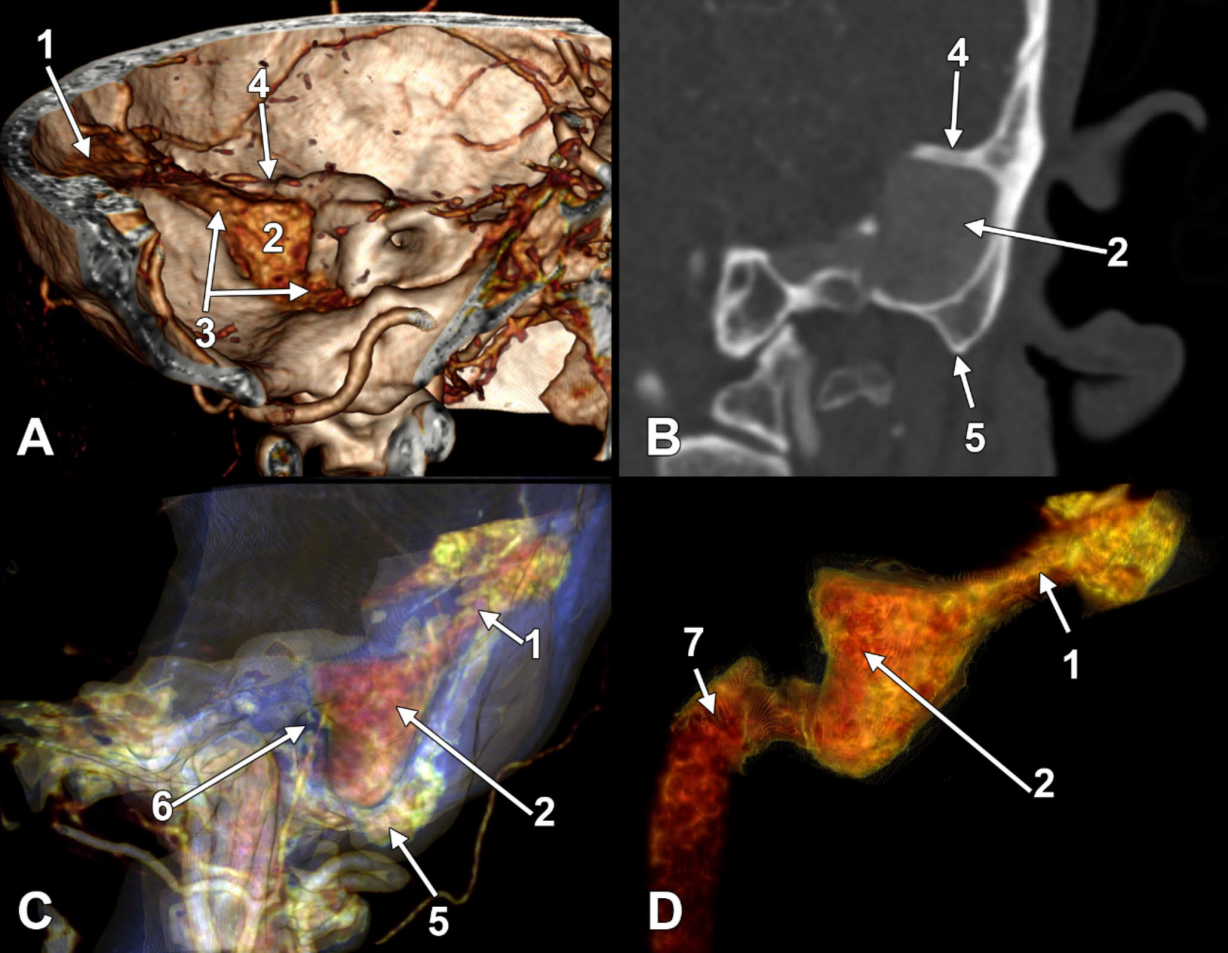
Mastoid pouch of the right sigmoid sinus in Case #2. (**A**) Three-dimensional volume rendering of the left side of the posterior cranial fossa, medial view. (**B**) Perpendicular two-dimensional section of the left temporal bone, anteriorly viewed. (**C**) Three-dimensional volume rendering of the left temporal bone with enhanced transparency, lateral view. (**D**) Three-dimensional rendering of the mastoid pouch of the left sigmoid sinus, postero-lateral view. (1) transverse sinus; (2) mastoid pouch of the sigmoid sinus; (3) sigmoid sinus; (4) tegmen sinus sigmoidei; (5) mastoid tip; (6) external auditory canal; (7) jugular bulb

**Fig. 2:**
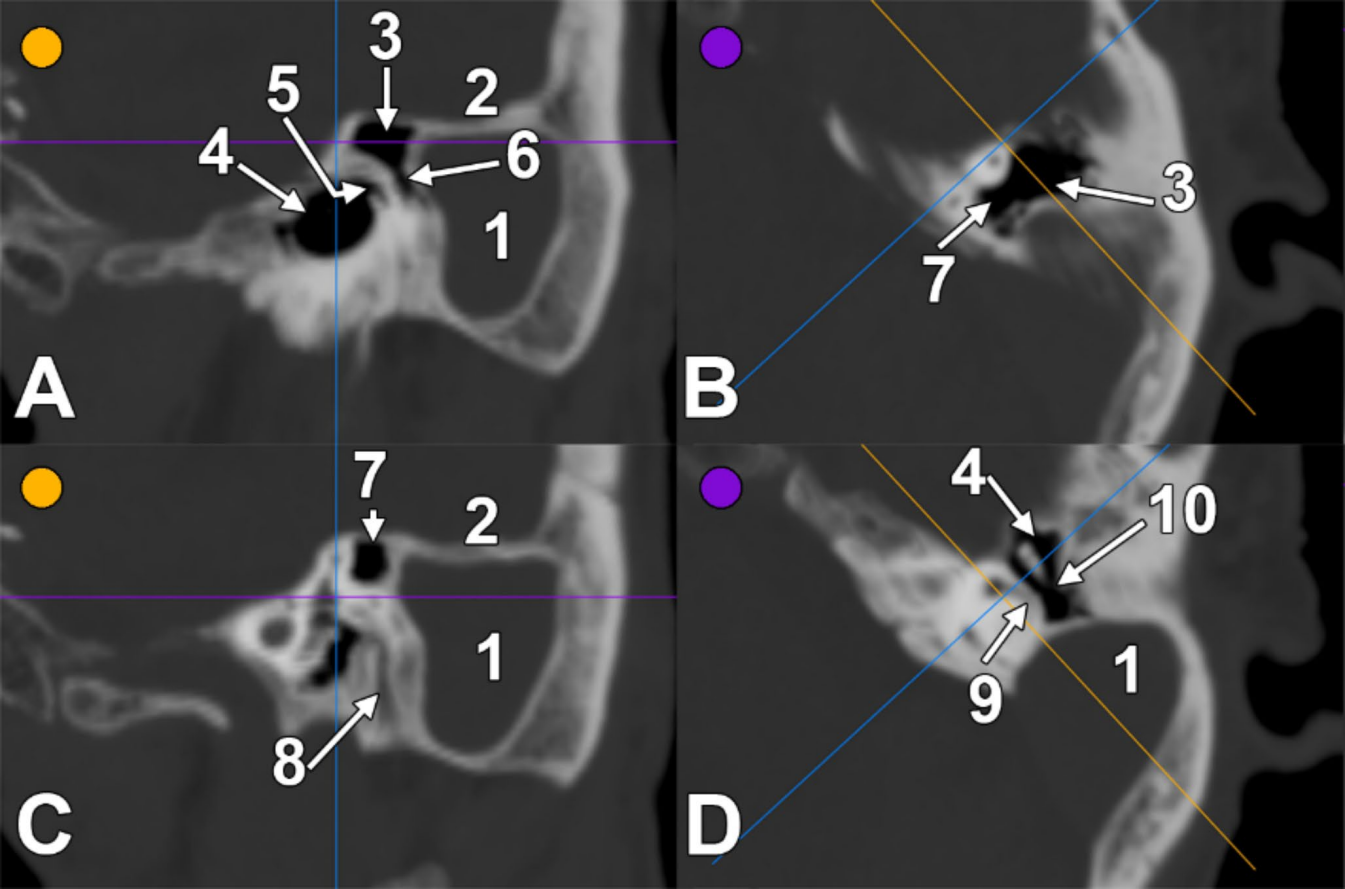
Series (**A**-**B** and **C**-**D**) of correlated two-dimensional sections of the right sigmoid sinus in Case #2. The guidelines indicate the levels of the sections. A, C. Laterally viewed sagittal slices through the sigmoid sinus pouch at the level of the mastoid antrum and tympanic cavity (**A**) and, respectively, of the facial canal (**C**). B, D. Inferiorly viewed axial slices through the medial recess of the mastoid antrum (**B**) and the aditus ad antrum (**D**). (1) mastoid pouch of the sigmoid sinus; (2) tegmen sinus sigmoidei; (3) mastoid antrum; (4) tympanic cavity; (5) lateral tympanic sinus; (6) inferior recess of the mastoid antrum; (7) medial recess of the mastoid antrum; (8) facial canal; (9) lateral semicircular canal; (10) aditus ad antrum

An outer pouch of 1.74 cm (largest transverse diameter) and 2.18 cm (largest sagittal diameter) of the left SS filled almost completely the mastoid cells’ anatomical space and reached the mastoid cortex laterally (**Online Resource 1**). That pouch of the SS (Figs. 1 and 2) was covered laterally by a tegmen sinus sigmoidei broad of 1.41 cm. In front of the SS pouch was a small antrum communicating anteriorly via the aditus ad antrum with the tympanic cavity and extending medially over the lateral semicircular canal and posterior to the superior one (Fig. 2). From that antrum, a narrow inferior recess long of 2.67 mm, with the transverse diameter of 2.55 mm and the sagittal diameter of 1.53 mm, descended at 1.41 mm lateral to the mastoid segment of the facial canal and posterior to the lateral tympanic sinus (Fig. 2). The SS pouch reached the mastoid cortex immediately posterior to the external auditory canal and MacEwen’s suprameatal triangle. The bony wall between the SS pouch and the mastoid portion of the facial canal was 4.65 mm thick (Fig. 2). There were no mastoid air cells on the inferior and lateral sides of the SS pouch. On the opposite side, the MACS was anatomically normal.

## Discussion

The application of opacity modulation of the skull bone to visualise intracranial structures such as dural sinuses during surgical planning is limited [3]. Adjusting the bones’ opacity to observe the posterior fossa’s venous anatomy simultaneously is particularly helpful for planning and guiding surgical approaches [3], mainly when extraordinary anatomical variants occur.

Mastoid cells, when present, are arranged systematically. This arrangement depends on the course and position of the SS relative to surrounding structures: the temporal squama cortex postero-superiorly, the mastoid cortex laterally and posteriorly, the medial margin of the mastoid tip inferiorly, and the posterior wall of the facial canal [6]. Meltzer (1934) classified the mastoid air cells into superior, antero-inferior and mesioposterior groups [6].

Although the isolated agenesis of the mastoid antrum is an extraordinary finding, the underdeveloped mastoid pneumatisation was sought due to chronic otitis media that determines a reactive osteogenic process that obliterates the MACS [10]. In such isolated agenesis of the mastoid antrum, the dura mater still lies high in its usual position, while the association of the uniformly low dura with only a thin plate of bone separating the bony meatus from the middle fossa dura indicates a congenital aetiology [10]. To the authors’ knowledge, when the agenesis of the mastoid antrum was found, an aberrant course of the SS was either not found or not reported. The antrum was found in the case reported here, although extremely small. The SS variant reached close to the antrum and the facial canal.

It was discussed a long time ago that the normal mastoid process has a spongy bone structure until the end of the first year, a partly pneumatised structure until the fifth year and complete pneumatisation after that period [7]. Although inflammatory conditions of the tympanic mucosa during the first months of life may switch the process of pneumatisation to one of sclerosis [7], this cannot be correlated with the finding in the second case where a strongly enlarged SS replaced the anatomical space commonly occupied by a pneumatised or a sclerotic mastoid.

An anteriorly displaced vertical segment of the SS is an important anatomical variation that triggered a debate about its causality [13]. The SS was found just underneath a one-millimetre bony flap in the posterior wall of the external auditory canal [13] or even underneath the skin of the posterior wall of the external auditory canal [2].

The SS location is variable and important during surgery within the mastoid cavity. Lateral skull-base approaches require a thorough understanding of the relationships of the SS to avoid intraoperative complications [12]. During a presigmoid retrolabyrinthine approach, drilling of the posterior temporal bone exposes the triangle of Trautmann, bordered by the SS. A mastoid pouch of the SS brings the sinus immediately beneath the external mastoid cortex and puts it at risk during the first drill. Such extraordinary variation restricts the working surgical space. A large mastoid pouch of the SS may impose a constrained surgical corridor in the retrofacial approach due to its lateralised position. Such a lateralised position however may expand the surgical field during a retrosigmoid approach.

SS diverticula have been linked to pulsatile tinnitus, with larger sizes reported in affected patients (6.21 ± 1.7 mm) compared to non-affected individuals (3.06 ± 1.38 mm) [1]. The mastoid pouch of the SS we report here measured 1.74 cm, replacing rather than protruding into the mastoid.

In conclusion, preoperative angioCT scans of patients are of great use in observing unexpected anatomical variations.

## Electronic supplementary material

Below is the link to the electronic supplementary material.

Supplementary Material 1

## Data Availability

No datasets were generated or analysed during the current study.
